# Gain-of-function microRNA screens identify miR-193a regulating proliferation and apoptosis in epithelial ovarian cancer cells

**DOI:** 10.3892/ijo.2013.1896

**Published:** 2013-04-15

**Authors:** HARUO NAKANO, YOJI YAMADA, TATSUYA MIYAZAWA, TETSUO YOSHIDA

**Affiliations:** 1 Biologics Research Laboratories, Kyowa Hakko Kirin Co., Ltd., Machida-shi, Tokyo 194-8533, Japan

**Keywords:** microRNA, cell proliferation, apoptosis, miR-193, ovarian cancer cell

## Abstract

MicroRNAs (miRNAs) are a small class of non-coding RNAs that negatively regulate gene expression, and are considered as new therapeutic targets for treating cancer. In this study, we performed a gain-of-function screen using miRNA mimic library (319 miRNA species) to identify those affecting cell proliferation in human epithelial ovarian cancer cells (A2780). We discovered a number of miRNAs that increased or decreased the cell viability of A2780 cells. Pro-proliferative and anti-proliferative miRNAs include oncogenic miR-372 and miR-373, and tumor suppressive miR-124a, miR-7, miR-192 and miR-193a, respectively. We found that overexpression of miR-124a, miR-192, miR-193a and miR-193b inhibited BrdU incorporation in A2780 cells, indicating that these miRNAs affected the cell cycle. Overexpression of miR-193a and miR-193b induced an activation of caspase 3/7, and resulted in apoptotic cell death in A2780 cells. A genome-wide gene expression analysis with miR-193a-transfected A2780 cells led to identification of ARHGAP19, CCND1, ERBB4, KRAS and MCL1 as potential miR-193a targets. We demonstrated that miR-193a decreased the amount of MCL1 protein by binding 3′UTR of its mRNA. Our study suggests the potential of miRNA screens to discover miRNAs as therapeutic tools to treat ovarian cancer.

## Introduction

MicroRNAs (miRNAs) are small non-coding RNAs of 20–22 nucleotides, and function to suppress the expression of target mRNAs by translation blockade and/or mRNA degradation (1,2). They are involved in many biological processes including cell proliferation, differentiation and apoptosis, and their dysregulation can contribute to the pathological state including cancer (1,3,4). Several groups have documented miRNA expression profiling in ovarian cancers using miRNA microarray and massive parallel sequencing technology (5–10). miR-93, miR-141, miR-200 and miR-214 are frequently upregulated whereas miR-100, miR-143, miR-145 and let-7 are downregulated in ovarian carcinomas compared with normal counterparts (5–9). Abnormal miRNA expression is due to DNA copy number amplification and deletion, epigenetic modification and/or the dysregualtion of miRNA processing in cancer state (7,11,12). miR-214 upregulated in ovarian cancer can target PTEN tumor suppressor gene whereas down-regulated let-7 can target the RAS oncogene (8,13), suggesting that miRNAs may have a role as novel class of oncogenes or tumor suppressor genes in ovarian cancer (14).

Based on these findings, the clinical potential of miRNAs as cancer biomarkers and/or therapeutic agents is widely recognized and accepted (15). A single miRNA can regulate multiple mRNA transcripts that cooperatively work in cellular differentiation and function (16–19). The use of miRNA mimics or anti-miRNAs may represent powerful therapeutic tools to accomplish regression and/or re-differentiation of cancer by effectively targeting tumor suppressive or oncogenic genes with less toxicity (15,20). Indeed, a number of pre-clinical trials of miRNAs are currently in progress (21). In this study, we performed a gain of function screen using miRNA mimics library containing 319 miRNAs to identify miRNAs that can affect cell proliferation in A2780 ovary cancer cells. We found several anti-proliferative miRNAs including miR-124, miR-192 and miR-193 in A2780, suggesting that the potential of miRNA screens for discovering miRNAs as therapeutic tools to treat ovarian cancer.

## Materials and methods

### Cell culture

Human ovarian cancer cell line A2780 was obtained from Dr T. Tsuruo (22), and human colorectal cancer cell line DLD-1 was obtained from the American Type Culture Collection (ATCC, Manassas, VA, USA). A2780 and DLD-1 were cultured in RPMI-1640 (Gibco, Life Technologies, Carlsbad, CA, USA) containing 50 IU/ml penicillin and 50 *μ*g/ml streptomycin (Gibco, Life Technologies), supplemented with 5% (A2780) or 10% (DLD-1) fetal bovine serum (FBS, JRH Biosciences, Lenexa, KS, USA) at 37°C in an atmosphere of 5% CO_2_.

### miRNA library screening

A gain of function miRNA screen on cell viability was performed using A2780 as previously described (23). A2780 was seeded at 2,500 cells per well in 96-well plates the day before transfection. Synthetic miRNA mimic library (human Pre-miR™ miRNA precursor library-ver.1, Ambion, Applied Biosystems, Foster City, CA, USA) was screened using 50 nM in a duplicate. The library contained 319 miRNAs registered in miRBase ver. 7.1 (http://www.mirbase.org/). miRNA mimics were transfected using Lipofectamine 2000 (Life Technologies) according to the manufacturer’s protocols. Pre-miR miRNA precursor molecule-negative control (13) (Ambion, Applied Biosystems) was used as a negative control for miRNA mimics. We confirmed transfection efficiency (>90%) using siControl TOX transfection control (50 nM, Dharmacon, Lafayette, CO, USA). After 3 days of transfection, the cell viability was measured using the Cell Titer-Glo Luminescent Cell Viability Assay (Promega, Madison, WI, USA) according to the manufacturer’s instructions. Data were expressed as percentage of the negative control. Several miRNA hits were selected to assess reproducibility and dose-dependency (5, 25 and 50 nM).

### BrdU incorporation assay

miRNA (25 nM)-transfected cells in 96-well format were harvested for one day, and then were incubated with 10 *μ*M of 5′-bromo-2-deoxy-uridine (BrdU) for 2–4 h. The cells were fixed with cold ethanol/HCl, and the incorporated BrdU was detected using BrdU labeling and detection kit III (Roche Diagnostics GmbH, Mannheim, Germany) according to the manufacturer’s instruction.

### Caspase 3/7 activation assay

miRNA (25 nM)-transfected cells in 96-well format were harvested for 2 days, and then used to measure caspase 3/7 activity using Caspase-Glo 3/7 assay (Promega) according to the manufacturer’s instruction.

### RNA isolation and whole genome microarray

A2780 cells were transfected with miR-193a (Pre-miR miRNA precursor molecules, hsa-miR-193a-3p, Ambion, Applied Biosystems) or negative control miRNA (25 nM), and allowed to grow in the medium (RPMI-1640) for 10 h before RNA isolation. Total RNA was isolated using the RNeasy mini RNA isolation kit (Qiagen). The integrity of the RNA was verified using an Agilent 2100 Bioanalyzer (1.8–2.0: Agilent Technologies, Palo Alto, CA, USA). Transcriptome microarray analysis was carried out using the 44K Whole Human Genome Microarray chip (Agilent Technologies) according to the manufacturer’s instructions. Scanning microarray chips and processing data were done by Pharmafrontier Co., Ltd, Kyoto, Japan. Differentially expressed probe sets were identified with a fold change >1.5. Gene ontology (GO) pathway enrichment analysis was performed among genes differentially expressed after miR-193a transfection by SigTerm software (24). The downregulated genes with miR-193a transfection were compared with predicted miR-193a target genes searched by TargetScan (http://www.targetscan.org/). Over-representation of predicted miR-193a target genes within downregulated gene sets was assessed by SigTerm software.

### Western blot analysis

miRNA or siRNA (25 nM)-transfected A2780 cells were lysed in radio immunoprecipitation assay (RIPA) buffer [50 mM Tris-HCl (pH 8.0), 150 mM sodium chloride, 1% NP-40, 0.5% sodium deoxycholate, 0.1% sodium dodecyl sulfate] supplemented with 1% of a protease inhibitor cocktail stock solution (set III, Roche Diagnostics GmbH) after 1 or 2 days transfection. The following pre-designed siRNA was used as a positive control: MCL1 siRNA (Hs_MCL1_6 HP validated siRNA, SI02781205, Qiagen GmbH, Hilden, Germany). Proteins (10 or 20 *μ*g) were separated by SDS-PAGE. Upon electroblotting to polyvinylidene fluoride (PVDF) membrane (Immobilon-P, Millipore, Billerica, MA, USA), non-specific binding sites were blocked by incubation in TBST (Tris-buffered saline/0.05% Tween-20) containing 1% skim milk, and then incubated with rabbit polyclonal anti-MCL1 (1:200, S-19, Santa Cruz Biotechnology Inc., Santa Cruz, CA, USA), or mouse monoclonal anti-α-tubulin (1:2,500, clone DM1A, Sigma, St. Louis, MO, USA) in blocking solution. After washing with TBST, the membrane was incubated with HRP-conjugated rabbit anti-mouse IgG secondary antibody (P0161, Dako, Glostrup, Denmark) or HRP-conjugated swine anti-rabbit IgG secondary antibody (P0217, Dako). Signals were detected using enhanced chemiluminescence (ECL) or ECL-plus reagent (Amersham™ GE Healthcare UK Ltd., Buckinghamshire, UK).

### qRT-PCR

Total RNA was prepared from miRNA or siRNA (25 nM)-transfected cells 2 days after transfection using RNeasy mini kit (Qiagen), and then first strand cDNA was synthesized using SuperScript III (Life Technologies) according to the manufacturer’s instruction. Real-time RT-PCR was performed using 7900 HT fast real-time PCR system (Applied Biosystems Inc., Foster City, CA, USA) with SYBR-Green as a reporter. The following primers were used for detection: MCL1 (222 bp) forward: TCTAAGTGCTGACTGGCTACG, reverse: CCTGGCACAGCTATCAAAAG; GAPDH (137 bp) forward: ACTTTGTCAAGCTCATTTCCTG, reverse: CTCTCTTCCTCTTGGCTCTTG.

### Luciferase miRNA target reporter assay

3′-untranslated regions (UTRs) of MCL1 gene (1546 bp), containing predicted binding sites of miR-193a, were amplified by PCR from A2780 cDNA, and inserted into the pGL3 control vector (Promega) by using Xba-I site immediately downstream from the stop codon of *Firefly* luciferase. The following primers were used: MCL1 3′-UTR forward: CGGCTAGCGAAAAGCAAGTGGCAAGAGG, reverse: CGGCTAGCAGGGAGGGTCACTCAGGTTT.

Deletion of the first 3 nucleotides corresponding miR-193a seed-region complementary site was inserted in mutant constructs using KOD-plus-Mutagenesis kit (Toyobo, Osaka, Japan), according to the manufacturer’s protocol. The following primers were used for generation of mutant constructs: MCL1-mutant-Primer 1: AGCCAGGCAAGTCATAGAATTGATT, MCL1-mutant-Primer 2: GGCCACTTTCCTGTTCTCAACAAGG.

DLD-1 cells were cultured in 96-well formats and co-transfected with 100 ng of pGL3 *Firefly* luciferase reporter vector, 20 ng of pRL-TK *Renilla* luciferase control vector (Promega) and 25 nM miRNA or negative control miRNA using Lipofectamine 2000. *Firefly* and *Renilla* luciferase activities were measured consecutively using the Dual-Luciferase Reporter Assay System (Promega) 24 h after transfection. All the experiments were done in triplicate and repeated at least twice on different days.

## Results

### Effects of miRNA mimic library transfection on cell proliferation of A2780 cell line

**Figure 1:**
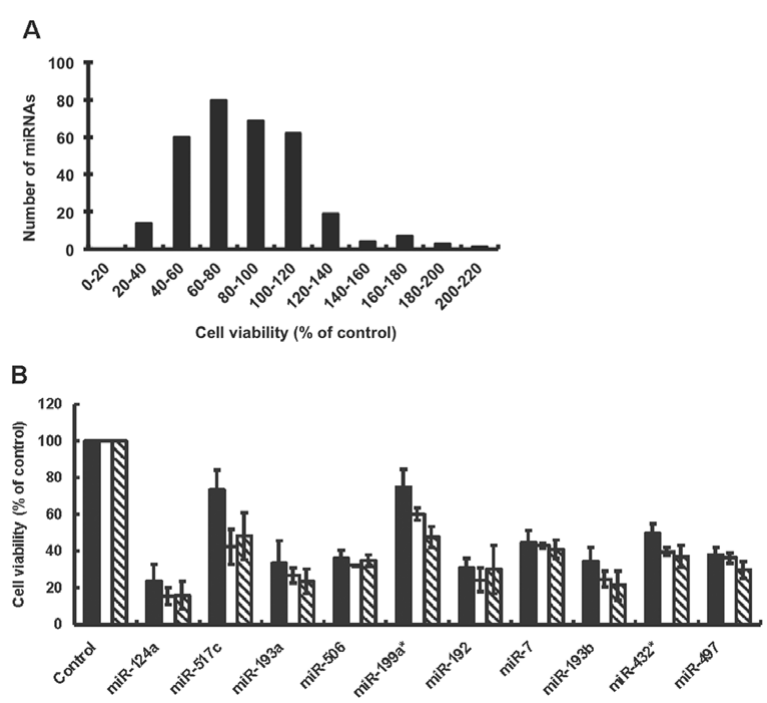
Gain of function screen of miRNA library in A2780 ovary cancer cell line. (A) A2780 cells were transfected with 319 miRNA mimics (50 nM) in duplicate, and their cell viability was measured 3 days after transfection. The graph shows the distribution of cell viability normalized by the miRNA mimic negative control. (B) Top 10 anti-proliferative miRNA mimics derived from 1st screen were transfected into A2780 at the final concentration of 5 nM (solid bar), 25 nM (open bar) and 50 nM (hatched bar). The cell viability was measured three days after miRNA mimic transfection. The data are normalized with the negative control and show average ± SD from three different experiments.

**Figure 2:**
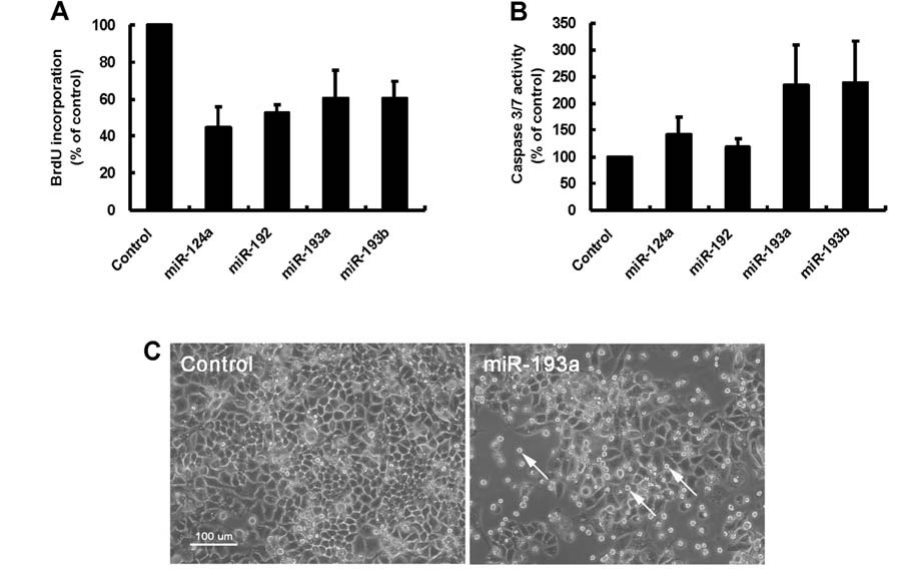
Effects of anti-proliferative miRNAs on DNA synthesis and caspase 3/7 activity. (A) Anti-proliferative miRNAs (miR-124a, miR-192, miR-193a and miR-193b) or negative control miRNA were transfected into A2780 at the final concentration of 25 nM. One day after transfection, the transfected cells were incubated with BrdU containing medium for 2 to 4 h, and then BrdU incorporation was measured. The data is normalized with the negative control and show average ± SD from three different experiments. (B) Anti-proliferative miRNAs (miR-124a, miR-192, miR-193a and miR-193b) or negative control miRNA were transfected into A2780 at the final concentration of 25 nM. Two days after transfection, caspase 3/7 activity in miRNA-transfected cells was measured. The data are normalized with the negative control and show average ± SD from three different experiments. (C) Cellular morphology of miR-193a (25 nM)-transfected A2780 cells. Arrows indicate apoptotic cell debris. Scale bar indicates 100 *μ*m.

To identify miRNAs that affect cell proliferation of ovarian cancer cells, we performed a gain of function screen using synthetic miRNA mimic library (319 miRNAs) for human epithelial ovary cancer cells (A2780). The library consists of miRNAs registered in early version of miRBase (ver. 7.1 in October, 2005, http://www.mirbase.org/), and many of them were expressed in ovarian normal and cancer tissues and cell lines (5). We detected cellular ATP to assess cell viability in miRNA (50 nM)-transfected cells 3 days after transfection. Frequency distribution indicated that broad ranges of miRNA mimic transfections affected the cell viability of A2780 (Fig. 1A). A total of 46 out of 319 miRNAs induced more than 50% changes in the cell viability of A2780 after 3 days transfection. Table I shows top 10 miRNAs that increased or decreased the cell viability of A2780. They included known oncogenic miRNAs such as miR-372 (cell viability, 187%) and miR-373 (165%), and tumor suppressive miRNAs such as miR-124a (28.3%), miR-7 (37.1%), miR-192 (36.6%) and miR-193a (29.7%) in several different cancer types (18,25–27). The seed family miRNAs that have the same sequences in seed region (2nd to 8th nucleotide) of miRNAs showed similar effects on cell viability in A2780 cells. For example, miR-93/miR-302/miR-372/mir-373 seed family miRNAs (miR-93, miR-302b, miR-302d, miR-372, miR-373) were pro-proliferative, while miR-193 seed family miRNAs (miR-193a, miR-193b) were anti-proliferative (Table I). miR-200/miR-141 seed family miRNAs that are upregulated in ovarian cancer (5,6,10) had a little effect on the cell viability in A2780 cells (the cell viability; 97.9, 113, 92.0 and 101% with miR-200a, miR-200b, miR-200c and miR-141 transfection, respectively). miR-100, miR-143 and miR-145 that are down-regulated miRNAs in ovarian cancer (5,6,8) induced a 15–30% decrease in the cell viability of A2780 (the cell viability; 84.1, 81.8 and 73.1 with miR-100, miR-143 and miR-145 transfection, respectively). We are interested in miRNA mimics that decreased the cell viability of A2780 since these miRNA mimics themselves could have therapeutic potential to treat ovarian cancer. To further evaluate miRNA mimics on the inhibition of cell proliferation in A2780, we selected top 10 anti-proliferative miRNAs (miR-7, miR-124a, miR-192, miR-193a, miR-193b, miR-199a^*^, miR-432^*^, miR-497, miR-506 and miR-517c) from the first screen, and examined the cell viability in A2780 cells transfected with different concentrations of miRNAs (5, 25, 50 nM). We confirmed results of our first screening at 50 nM, and found that the transfection of miR-124a, miR-192, miR-193a and miR-193b induced a large decrease in the cell viability of A2780 even at 5 nM (Fig. 1B), indicating that these miRNAs had a profound anti-proliferative effect in A2780 cells. We examined whether miR-124a, miR-192, miR-193a and miR-193b affected DNA synthesis to inhibit cell proliferation in A2780 cells. One day after miRNA transfection, BrdU incorporation was examined to evaluate DNA synthesis in transfected cells. As shown in Fig. 2A, miR-124a, miR-192, miR-193a and miR-193b decreased an incorporation of BrdU compared with the negative control, indicating that these miRNAs induced the inhibition of DNA synthesis in A2780 cells. We next examined whether these miRNAs affected apoptotic pathway to inhibit cell proliferation in A2780 cells. We found that miR-193a and miR-193b but not miR-124a and miR-192 induced more than twofold increase in an activity of caspase 3/7, the effector of apoptotic pathway, in A2780 cells (Fig. 2B). The result indicated that miR-193a and miR-193b could induce the apoptotic cell death in A2780 cells. Actually, apoptotic cell debris was frequently observed in miR-193a-transfected A2780 cells (Fig. 2C, arrows).

### Transcriptome analysis to assess target genes regulated by miR-193a

**Figure 3:**
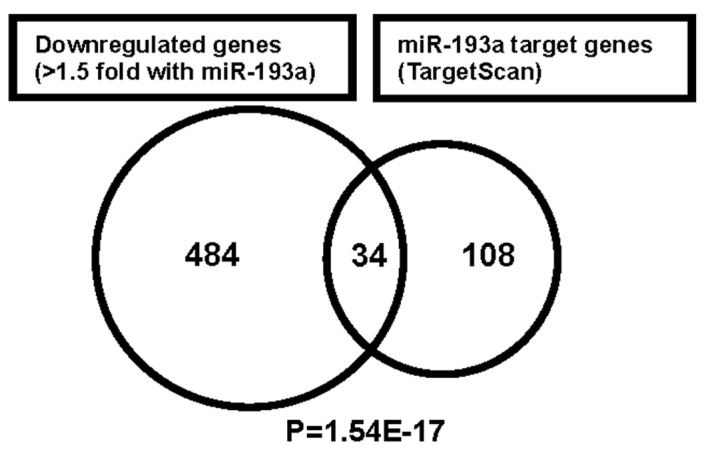
Transcriptome analysis with miR-193a-transfected A2780. Venn diagram to illustrate the relationship between the downregulated genes (10 h after miR-193a transfection) and predicted target genes by TargetScan. Predicted miR-193a target genes within the downregulated gene sets were significantly enriched by SigTerm software (P=1.54E-17).

We further characterized the anti-proliferative effect of miR-193a in A2780 cells. To examine target genes regulated by miR-193a, we performed genome wide gene expression analysis using miR-193a (25 nM)-transfected cells compared with the negative control miRNA-transfected ones. We identified 518 genes that were downregulated more than 1.5-fold by miR-193a transfection after 10 h. To evaluate the potential functional significance of the genes downregulated after miR-193a transfection, we subjected the gene expression data to gene ontology (GO) pathway enrichment analysis. The 20 most significantly over-represented pathways listed in Table II include small GTPase signaling and vesicular transport. We compared these downregulated 518 genes with predicted miR-193a target genes (142 genes) obtained by TargetScan (Fig. 3). This resulted in the match of 34 candidate miR-193a target genes, and they were significantly over-represented in the downregulated gene sets by using the SigTerm software (24). Table III showed 34 candidate miR-193a target genes obtained by our transcriptome analysis. The candidate genes include ARHGAP19 (RhoGAP19), CCND1 (cyclin D1), ERBB4, KRAS and MCL1 that function in cell signaling, cell cycle and apoptotic pathway.

### MCL1 is a direct target gene of miR-193a in A2780 cells

**Figure 4:**
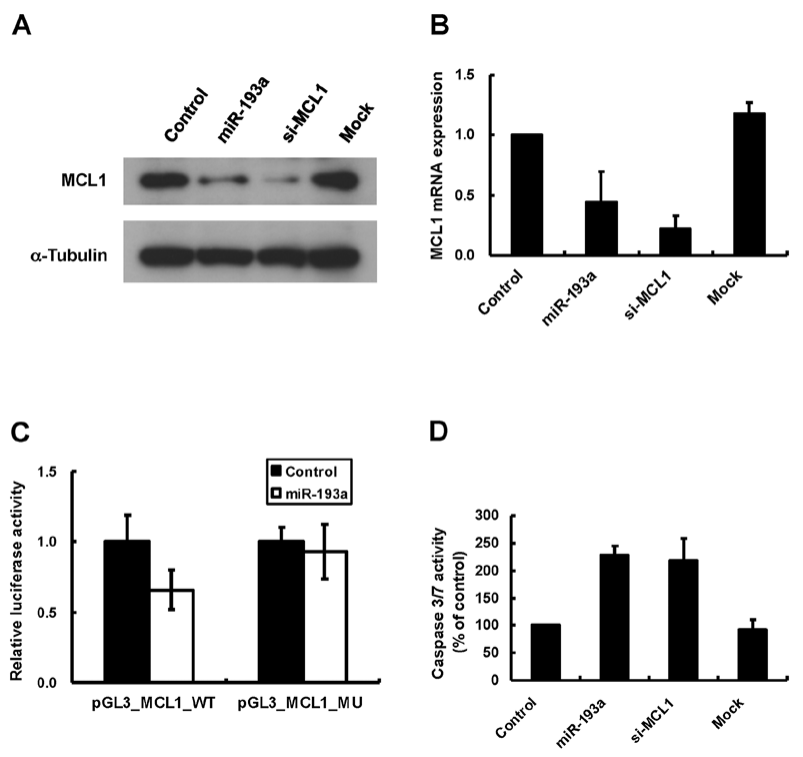
miR-193a targets the anti-apoptotic factor MCL1. (A) Immunoblot analysis for detection of MCL1 with anti-MCL1 antibody. Cell lysate with miR-193a or MCL1 siRNA (25 nM)-transfected cells was collected two days after transfection. Total protein (10 *μ*g) was loaded for analysis. The immunodetection of α-tubulin was used as loading control. The representative blots of three different experiments are shown. (B) Relative quantification of MCL1 expression by qRT-PCR in miR-193a or MCL1 siRNA (25 nM)-transfected cells two days after transfection. The data are normalized by GAPDH expression, and expressed as relative values against the negative control. Average ± SD from three different experiments are shown. (C) Reporter assay showing decreased luciferase activity in DLD-1 cells co-transfected with pGL3-MCL1-3′UTR (pGL3_MCL1_WT) and miR-193a (25 nM, open bar). Deletion of miR-193a seed region complementary site from the 3′UTR (pGL3_MCL1_MU) attenuated miR-193a-induced decrease of luciferase activity. *Firefly*/*Renilla* luciferase activity is expressed as relative values against the negative control. Average ± SD (n=3) from one representative experiment are shown. (D) Caspase 3/7 activity of miR-193a or MCL-1 siRNA (25 nM)-transfected cells was measured 2 days after transfection. The data show average ± SD from three different experiments.

From our results of transcriptome analysis, we focused on MCL1 gene as miR-193a targets, since MCL1 was an anti-apoptotic gene of BCL2 family (28), and therefore might contribute to miR-193a-induced cell death in A2780 cells. MCL1 3′UTR contains one potential target site of miR-193a and the site is conserved between human and mouse. To examine the regulation of miR-193a on MCL1 protein expression, we performed western blot analysis with miR-193a-transfected A2780 cells. Transfection of positive control MCL1 siRNA induced the decrease in endogenous MCL1 proteins in A2780 cells (Fig. 4A). We demonstrated that overexpression of miR-193a decreased MCL1 proteins in A2780 cells (Fig. 4A). We next performed qRT-PCR with miR-193a-transfected cells to examine whether miR-193a affected MCL1 mRNA expression. We found that miR-193a induced about 50% decrease in MCL1 mRNA expression in A2780 cells (Fig. 4B). These results indicated that miR-193a affected MCL1 expression at both protein and mRNA levels. To validate whether miR-193a can directly regulate the translation of MCL1 mRNAs, we constructed a luciferase reporter plasmid that inserted MCL1 3′UTR (around 1.5 kb) at the downstream of *Firefly* luciferase gene, and tested the luciferase activity. As shown in Fig. 4C, co-transfection of miR-193a and MCL1 3′UTR reporter vector induced around 40% reduction of the luciferase activity compared with co-transfection of the negative control miRNA and the reporter vector. The decrease of the luciferase activity was attenuated by using the mutant reporter vector deleting miR-193a seed region complementary sites in MCL1 3′UTR (Fig. 4C, MCL1-3′UTR-MU). These results indicated that MCL1 would be a direct target of miR-193a. We further examined whether the downregulation of endogenous MCL1 could induce apoptosis in A2780 cells. As shown in Fig. 4D, the transfection of MCL1 siRNA (25 nM) induced caspase 3/7 activation comparable with miR-193a transfection in A2780 cells (Fig. 4D), indicating that the downregulation of MCL1 by miR-193a could contribute to miR-193a-induced apoptosis in A2780 cells.

## Discussion

Several studies reveal that global miRNA expression is dysregulated in ovarian cancer (5–10), and miRNAs may represent new targets for detection, diagnosis and therapy in ovarian cancer (14). However, functions of many miRNAs in ovarian cancer remain to be elucidated. In this study, we performed a gain-of-function screen using a miRNA mimic library (319 miRNA species) to identify those affecting cell proliferation in epithelial ovarian cancer cells (A2780). The library consists of miRNAs registered in early version of miRBase (ver. 7.1 in October, 2005, http://www.mirbase.org/), and many of them were expressed in ovarian normal and cancer tissues and cell lines (5). We discovered pro-proliferative miRNAs (miR-9^*^, miR-93, miR-130a, miR-130b, miR-301, miR-302b, miR-302d, miR-363, miR-372, miR-373), and anti-proliferative miRNAs (miR-7, miR-124a, miR-192, miR-193a, miR-193b, miR-199a^*^, miR-432^*^, miR-497, miR-506, miR-517c) in A2780 cells. By the same miRNA mimics library screening, we found that miR-93/miR-372/miR-373 and miR-124a were pro-proliferative and anti-proliferative, respectively, in DLD-1 colorectal cancer cells (23), suggesting consistent roles of these miRNAs on cell proliferation in ovary and colorectal cancer cells. The base-pairing between target mRNAs and the seed region (2nd to 8th nucleotides) of miRNA is important for miRNAs to function to regulate their target genes (2). The seed family miRNAs induced similar cellular phenotypes on cell proliferation in this study (ex. pro-proliferative miR-93, miR-302b, miR-302d, miR-372, miR-373 and anti-proliferative miR-193a, miR-193b), supporting the importance of the seed region of miRNA on its function. Our miRNA hits did not always correspond to dysregulated miRNAs reported in ovarian cancer (5–10), but included pro-proliferative miR-93 that was upregulated in primary ovarian carcinomas (6), supporting an oncogenic role of this miRNA in ovarian cancer. Our miRNA hits also included tumor suppressive miR-7, miR-124a, miR-192 and miR-193a in several cancer types (18,25–27), suggesting that these miRNAs could be tumor suppressive in ovarian cancer. Among our miRNA hits, we further characterized miR-124a, miR-192, miR-193a and miR-193b that induced a large decrease in the cell viability of A2780 cells. miR-124a and miR-192 induced a decrease in BrdU incorporation, indicating that these miRNAs affected cell cycle resulting in inhibition of DNA synthesis in A2780 cells. Inhibitory effects of miR-124a and miR-192 on cell cycle gene pathway are reported in several cancer cell lines. miR-124a targets cyclin dependent kinase 6 (CDK6), and thereby inhibits the phosphorylation of retinoblastoma (Rb) in HCT116 cells (29). miR-192 is upregulated by genotoxic stress in HCT116, A549 and U2OS cell lines bearing wild-type p53, and induces the cell cycle arrest by enhancing CDKN1A/p21 expression (18,30).

We showed that miR-193a and miR-193b inhibited BrdU incorporation and induced caspase 3/7 activation in A2780 cells, indicating that these miRNAs could affect cell cycle and apoptotic gene pathways. Our transcriptome analysis with miR-193a-transfected A2780 cells identified ARHGAP19, CCND1, ERBB4, KRAS, MCL1 as potential miR-193a target genes. We demonstrated that the translation of MCL1 proteins was suppressed by miR-193a, suggesting that anti-apoptotic MCL1 would be one of the target genes for miR-193a-induced cell death in A2780. Anti-proliferative and pro-apoptotic functions of miR-193 are reported in several cancer cell lines including MDA-MB-453 (breast cancer), Malme-3M, SKMEL-28, SKMEL-5 (melanoma), HO-1-N-1, HSC-2 (oral squamous cell carcinoma), 22Rv1 (prostate cancer), SK-Hep-1 (hepato-cellular carcinoma) and Kasumi-1 (acute myeloid leukemia) (26,31–36). Consistent with our results, CCND1, KRAS and MCL1 are identified as miR-193 target genes (26,32,33,37). miR-193a gene locus (chromosomal region 17q11.2) has CpG islands that are hyper-methylated in oral cancer (26) and acute myeloid leukemia (36) compared with normal tissues and cells. miR-193a is downregulated in epithelial ovary cancer compared with normal counterparts (10), but study is needed on whether the miR-193a gene locus is hyper-methylated in ovary cancer.

Exogenous expression of a single miRNA mimic can coordinately regulate gene expression on cellular function, which encourages the therapeutic use of miRNA to direct cancer cell death and/or re-differentiation without undesirable side-effects (15,20). One of the challenges to the therapeutic use of miRNA is to predict precisely molecular consequences induced by modulating cellular miRNAs. Transcriptome analysis by microarray has been widely used for miRNA target identification at the transcription level. Protein-profiling techniques have been applied to miRNA-transfected cells for the identification of miRNA targets at the translational level (38–41).

In summary, we performed a gain-of-function miRNA screen and discovered several miRNAs affecting cell proliferation and death in A2780 ovary cancer cells. Among them, we identified miR-193a as strong anti-proliferative miRNAs in A2780 cells. miR-193a induced the inhibition of DNA synthesis and apoptosis by targeting genes including ARHGAP19, CCND1, ERBB4, KRAS, MCL1, indicating a tumor suppressive role of this miRNA in epithelial ovarian cancer cells. Our study suggests the potential of miRNA screens to discover miRNAs as therapeutic tools to treat ovarian cancer.

## Figures and Tables

**Table I:** Results of miRNA library screening.

miRNAs that increased cell viability		miRNAs that decreased cell viability	
	
miRNA	Cell viability (%)	miRNA	Cell viability (%)
miR-301	218	miR-124a	28.3
miR-372	187	miR-517c	29.4
miR-93	185	miR-193a	29.7
miR-302b	181	miR-506	31.9
miR-130a	173	miR-199a^*^	34.5
miR-302d	172	miR-192	36.6
miR-363	166	miR-7	37.1
miR-373	165	miR-193b	37.7
miR-9^*^	162	miR-432^*^	37.8
miR-130b	162	miR-497	38.3

Data represent the cell viability in miRNA mimics (50 nM)-transfected cells 3 days after transfection. Data were expressed as a percentage of the negative control in an average of duplicates. Top 10 miRNAs that increased or decreased the cell viability are listed.

**Table II:** Twenty most significantly enriched (P<0.05) gene ontology (GO) pathways among downregulated genes after miR-193a transfection into A2780 cells.

Term	P-value
Small GTPase regulator activity	0.0022
Ras guanyl-nucleotide exchange factor activity	0.0030
Rho guanyl-nucleotide exchange factor activity	0.0052
Regulation of Rho protein siganal transduction	0.0060
Blood vessel development	0.0069
Cytoplasmic vesicle part	0.0072
Vasculature development	0.0080
Post-Golgi vesicle-mediated transport	0.0080
Protein localization	0.0149
Phospholipid transporter activity	0.0155
Regulation of small GTPase mediated signal transduction	0.0156
Guanyl-nucleotide exchange factor activity	0.0168
GTPase regulator activity	0.0181
Macromolecule localization	0.0187
Insulin receptor signaling pathway	0.0193
Guanylate kinase activity	0.0196
Early endosome	0.0213
Neuron projection	0.0217
Intracellular signaling cascade	0.0225
One-carbon compound metabolic process	0.0229

**Table III:** Candidate miR-193a target genes downregulated in miR-193a-transfectants.

Entrez gene ID	Symbol	Fold change
23119	HIC2	−6.20
10152	ABI2	−3.34
595	CCND1	−3.30
54756	IL17RD	−3.20
10238	WDR68	−3.15
3925	STMN1	−2.97
5324	PLAG1	−2.97
3845	KRAS	−2.76
4076	CAPRIN1	−2.66
2066	ERBB4	−2.58
57704	GBA2	−2.45
84986	ARHGAP19	−2.23
10620	ARID3B	−2.17
7342	UBP1	−2.09
27242	TNFRSF21	−2.07
4170	MCL1	−2.00
56262	LRRC8A	−1.93
10160	FARP1	−1.91
57472	CNOT6	−1.91
23179	RGL1	−1.79
23341	DNAJC16	−1.79
88455	ANKRD13A	−1.70
4215	MAP3K3	−1.67
114991	ZNF618	−1.66
23492	CBX7	−1.64
23365	ARHGEF12	−1.64
22883	CLSTN1	−1.61
9939	RBM8A	−1.60
54890	ALKBH5	−1.59
115	ADCY9	−1.57
4189	DNAJB9	−1.51
1173	AP2M1	−1.51
9962	SLC23A2	−1.51
23384	SPECC1L	−1.50
